# Machine learning with in silico analysis markedly improves survival prediction modeling in colon cancer patients

**DOI:** 10.1002/cam4.5420

**Published:** 2022-11-07

**Authors:** Choong‐Jae Lee, Bin Baek, Sang Hee Cho, Tae‐Young Jang, So‐El Jeon, Sunjae Lee, Hyunju Lee, Jeong‐Seok Nam

**Affiliations:** ^1^ School of Life Sciences Gwangju Institute of Science and Technology Gwangju Korea; ^2^ School of Electrical Engineering and Computer Science Gwangju Institute of Science and Technology Gwangju Korea; ^3^ Department of Hemato‐Oncology Chonnam National University Medical School Gwangju Korea; ^4^ Cell Logistics Research Center Gwangju Institute of Science and Technology Gwangju South Korea

**Keywords:** biomarkers, clinical outcome, colon cancer, in silico system analysis, machine learning, survival prediction model

## Abstract

**Background:**

Predicting the survival of cancer patients provides prognostic information and therapeutic guidance. However, improved prediction models are needed for use in diagnosis and treatment.

**Objective:**

This study aimed to identify genomic prognostic biomarkers related to colon cancer (CC) based on computational data and to develop survival prediction models.

**Methods:**

We performed machine‐learning (ML) analysis to screen pathogenic survival‐related driver genes related to patient prognosis by integrating copy number variation and gene expression data. Moreover, in silico system analysis was performed to clinically assess data from ML analysis, and we identified *RABGAP1L*, *MYH9*, and *DRD4* as candidate genes. These three genes and tumor stages were used to generate survival prediction models. Moreover, the genes were validated by experimental and clinical analyses, and the theranostic application of the survival prediction models was assessed.

**Results:**

*RABGAP1L*, *MYH9*, and *DRD4* were identified as survival‐related candidate genes by ML and in silico system analysis. The survival prediction model using the expression of the three genes showed higher predictive performance when applied to predict the prognosis of CC patients. A series of functional analyses revealed that each knockdown of three genes reduced the protumor activity of CC cells. In particular, validation with an independent cohort of CC patients confirmed that the coexpression of *MYH9* and *DRD4* gene expression reflected poorer clinical outcomes in terms of overall survival and disease‐free survival.

**Conclusions:**

Our survival prediction approach will contribute to providing information on patients and developing a therapeutic strategy for CC patients.

## 1 INTRODUCTION

Colon cancer (CC) is one of the most frequently diagnosed cancers and a leading cause of cancer‐related death worldwide. CC patients, even those with the same disease stage, have different survival outcomes according to molecular characteristics related to their genetic and environmental factors. To understand such tumor heterogeneity, prognostic markers need to be developed and used in treatment strategies. Thus, further development of prognostic models integrating multiple prognostic markers may help to optimize individualized clinical decision‐making regarding adjuvant treatment for those at higher risk of mortality, contributing to the successful treatment of CC.

Survival prediction models are designed to assist treatment decision‐making by predicting the patient's risk class, diagnosis, prognosis, and recurrence risk according to information on individual patients.^1^ To make predictions, large and complex patient data sets should be analyzed to identify important patient characteristics and classify patients accordingly.^2^, ^3^, ^4^, ^5^ Machine learning (ML) has recently been widely used in the field of biology to develop survival prediction models; ML can facilitate classification, feature selection, and prediction by analyzing large, complex data.^4^, ^5^, ^6^ ML can conduct self‐learning by using diverse algorithms to develop survival prediction models. The survival prediction model interprets patient characteristics and predicts patient prognosis by using genomic biomarkers, including mutation, copy number, and gene expression, which are identified based on computational data.^7^, ^8^ Recently, many cancer studies have used ML to discover biomarkers in various cancers, such as CC, pancreatic cancer, and liver cancer, and to develop survival prediction models using biomarkers from ML.^9^, ^10^ However, it remains a challenge whether survival prediction models based on ML can achieve high prognostic performance when applied clinically.

In this study, we identified survival‐related genes by integrating copy number variation (CNV) and gene expression data by ML and performed further validation by in silico system analysis based on clinical genomic data. Then, *RABGAP1L*, *MYH9*, and *DRD4* were identified. Using these three genes and tumor stage information, we built a survival prediction model. In parallel, a series of functional analyses were conducted to verify that the three genes facilitated malignant behaviors in CC cells, and clinical validation with an in‐house cohort of CC patients validated that the genes were related to poor survival outcomes. This study provides new prognostic biomarkers and insights into the development of survival prediction models.

## 2 MATERIALS AND METHODS

### 2.1 Data preprocessing

#### 2.1.1 Data sources

Ribonucleic acid sequencing (RNA‐seq) gene expression and CNV data and clinical information were downloaded from the TCGA‐COAD project. This data set is publicly available on the Genomic Data Commons Data Portal (https://portal.gdc.cancer.gov/). The RNA‐seq data were sequenced using an Illumina HiSeq 2000 system; the expression levels are expressed herein as fragments per kilobase per million sequenced reads. The deoxyribonucleic acid (DNA) CNV data were obtained from Affymetrix single nucleotide polymorphism (SNP) 6.0 arrays, and the data type is the copy number segment. The averaged log2 ratios of CNVs in each segment are given with their associated contiguous chromosome regions in a tab‐delimited format. Clinical information was collected from cBioPortal for Cancer Genomics (http://www.cbioportal.org/).

#### 2.1.2 CNV preprocessing

As the CNV data were given as locus information, chromosomal regions were annotated using the Human Gene Organization (HUGO) Gene Nomenclature Committee gene symbols^11^ to allow for systematic comparison with expression data. If a gene matched multiple probes, it was given a value equal to the sum of the segment mean, multiplied by the proportion of each segment (refer to Figure S1 for details). After this procedure, genes with zero values in all samples or those with “not available (NA)” values in more than 10% of their samples were excluded. The remaining missing values were replaced with average values from other samples within the same gene. Subsequently, GISTIC 2^12^ was applied to the CNV data using the *Homo sapiens* (hg38) reference sequence gene annotation. CNV values were obtained by examining the distribution of log2 ratios to identify peaks related to CNV status. Default GISTIC log2 thresholds (0.1% and −0.1%) were used to identify gains and losses of genes with focal CNV changes.

#### 2.1.3 Gene expression preprocessing

The Ensembl IDs of gene expression data were remapped to gene symbols with the package “biomaRt” (ver. 2.40.5) in R; no coding genes were removed from the data. To isolate differentially expressed human protein‐coding genes, the “DESeq2” (ver. 1.24.0) package^13^ was used in R v.3.6.3. Genes with a value of zero in all normal samples were removed.

### 2.2 Calculation of candidate driver gene scores

The dominant effect of the cancer driver genes (DEOD) method was previously developed to measure the potential effects of driver genes across an entire network.^14^ For each gene with focal copy number changes, DEOD was used to estimate weights from CNVs in relation to the expression changes of its neighboring genes. It was then used to calculate a driver score for the gene in question. Here, DEOD was applied to the preprocessed CNV and gene expression data from TCGA‐COAD to obtain the driver scores for the candidate cancer driver genes. In this process, the human protein–protein interaction network, which has 8549 reference proteins and 362,553 interactions, was obtained from BioGRID (vHomo_sapiens_3_5.187. Table 3); these data were also used as inputs for the DEOD method.

### 2.3 ML‐based survival analysis

To determine how the CNVs or expression of the candidate cancer driver genes affected the clinical prognosis of patients with colorectal cancer (CRC), Kaplan–Meier survival curves were plotted for overall survival (OS) and disease‐free survival (DFS) in each of the amplification and deletion groups. The detailed methods are provided in Supplementary Method 1.1.

### 2.4 Regression analysis of expression profiles

#### 2.4.1 In‐house test data set for prediction models

A total of 137 patients with stage II and III colon cancer after curative surgical resection from Jan 2013 to Dec 2014 were included in this study. Normal and tumor tissue samples from each patient were provided by the Biobank of Chonnam National University Hwasun Hospital, a member of the Korea Biobank Network, with informed consent. This study was approved by the Chonnam National University Hwasun Hospital Institutional Review Board (approval number: IRB CNUHH‐2020‐173) and conducted in accordance with the Declaration of Helsinki. The clinical information on the 137 patients is shown in Table S1. This data set is referred to as Chonnam‐COAD.

#### 2.4.2 Public cohorts of metastatic CRC patients

To verify the robustness and stability of our prediction models, two public data sets for CRC patients (GSE17536 and GSE17537) were used. GSE17536 has 177 patients, with 73 deceased patients and 36 recurrence patients. GSE17537 has 55 patients, with 20 deceased patients and 19 recurrence patients. The gene expression and clinical information on the public cohorts GSE17536 and GSE17537 were downloaded from the Gene Expression Omnibus (GEO) database (https://www.ncbi.nlm.nih.gov/geo/).

#### 2.4.3 Logistic regression models for predicting patient prognosis

We developed logistic regression models for predicting patient prognosis as previously described.^8^ The TCGA‐COAD data set was used to train logistic regression models for the prediction of clinical prognosis (OS and DFS) for cancer patients. A logistic regression algorithm is a statistical model that understands relationships between variables and is a generalized linear model that can be used when the dependent variable is binary. The logistic regression model predicts an outcome based on some predictor variable, so the formula follows Equation 1.

(1) $$\log\left(\frac{p}{1-p}\right)=\beta_{1}X_{1}+\beta_{2}X_{2}+\cdots +\beta_{0}.$$

The input features ($X_{1},X_{2},\cdots$) in Equation (1) included combinations of expression profiles of the three selected cancer‐driver candidate genes and American Joint Committee on Cancer (AJCC) tumor stages. As the OS and DFS prediction models yield prediction probabilities, the results needed to be classified as binary values (alive/dead and disease‐free/recurrent for OS and DFS, respectively). Prior to the analysis, all cohorts, including TCGA‐COAD, Chonnam‐COAD, GSE17536, and GSE17537, were rescaled according to Equation 2:

(2) $$x_{\mathrm{ij}}^{\prime }=\frac{x_{\mathrm{ij}}-{\bar{x}}_{j}}{\sigma_{j}},$$

where ${x_{\mathrm{ij}}}^{\prime }$ represents sample *i* with gene *j*, ${\bar{x}}_{j}$ is the arithmetic mean of gene *j*, and $\sigma_{j}$ is its standard deviation (SD).

The clinical prognosis was predicted using logistic regression analysis of gene expression; this process was divided into three steps. In the first step, logistic regression models with different feature combinations were fitted with the TCGA‐COAD data set. In the second step, the model was tested on the Chonnam‐COAD data set and three other public data sets. Finally, the predicted probability was assessed with four statistical metrics: area under the curve (AUC), F1 score (F1), precision (Prec), and sensitivity (Sens). When the F1 score was the highest, the corresponding probability value was set as the threshold to divide the predicted probability scores into binary labels.

#### 2.4.4 Comparison methods

To further evaluate the performance of the three selected genes for predicting prognosis in CRC patients, we built two additional predictive models using the larger numbers of genes and compared the performance outcomes of the models. The first model used candidate driver genes identified by DEOD, and the second model used DEOD input genes. Because both models consider a large number of genes, a dimension reduction process was applied using a three‐layer autoencoder. An autoencoder is an artificial neural network architecture that aims to learn how to reconstruct input data. The autoencoder consists of an encoder and a decoder and an embedding layer that connects them. The encoder reaches the embedding layer while reducing the dimension of the input data, and the decoder expands the reduced embedding layer using data with the same dimension as the input data such that the output of the decoder becomes similar to the input of the encoder. In this study, both employ a rectified linear unit for their activation function and mean squared error as their loss function. The hyperparameter was used as the best‐case among several combinations of trials (epochs 2000, learning rate 0.0001, weight decay 0.0001, and drop rate 0.1). Then, logistic regression with reduced variables and tumor stage was used to predict survival. All predictive models in this subsection were trained and tested with stratified five‐fold cross‐validation on 223 patients from TCGA‐COAD.

### 2.5 Cell culture

The human CC cell line HCT116 was obtained from the Korean Cell Line Bank (Seoul, Republic of Korea) and grown in RPMI‐1640 (Welgene, Daegu, Republic of Korea) supplemented with 5% fetal bovine serum (Welgene) and 1% penicillin/streptomycin (Welgene) at 37 °C in a 5% CO_2_ incubator.

### 2.6 Knockdown of target genes

Small interfering RNAs (siRNAs) were purchased from Bioneer (Daejeon, Republic of Korea). siRNA transfection was performed using NEPA21 (Nepa Gene, Shioyaki, Japan). The electroporation parameters for HCT116 cells were the following: voltage 175 V, pulse length 50 ms, pulse interval 50 ms, and a number of pulses 5. Knockdown efficiencies were measured by RT‐qPCR and Western blotting. The siRNA sequences are listed in Table S2.

### 2.7 RNA isolation and real time‐quantitative polymerase chain reaction (RT‐qPCR)

RNA isolation from the cell lines and RT‐qPCR were conducted as previously described.^15^ The detailed methods are provided in the online supplement.

For RNA isolation from patient tissue, total RNA was extracted using Hybrid‐RTM (GeneAll Biotechnology). cDNA synthesis was performed using GoScript Reverse Transcription Mix (Promega) according to the supplier's instructions. Real‐time qPCR was conducted using a Bio‐Rad CFX96 Connect Real‐Time PCR Detection System (Bio‐Rad). The relative mRNA expression of selected genes was normalized to β‐actin. The sequences of the primers are listed in Table S3.

### 2.8 Protein isolation and western blotting

Protein isolation and Western blot analysis were conducted as previously described.^15^ The detailed methods are provided in the online supplement. The antibodies used for the Western blot assay are listed in Table S4.

### 2.9 Cell viability assay

Cell viability assays were conducted using thiazolyl blue tetrazolium bromide (MTT, Sigma–Aldrich) according to the manufacturer's instructions. The detailed methods are provided in the online supplement.

### 2.10 Clonogenic assay

The clonogenic assay was conducted as previously described.^15^ The detailed methods are provided in the online supplement.

### 2.11 Apoptosis assay

Quantitative analysis of the apoptotic cells was performed as previously described.^15^ The detailed methods are provided in the online supplement.

### 2.12 In vitro limiting dilution assay

An in vitro limiting dilution assay was performed as previously described with slight modifications. The detailed methods are provided in the online supplement.

### 2.13 Wound healing assay

A wound healing assay was performed as previously described with slight modifications. The detailed methods are provided in the online supplement.

### 2.14 Statistical analysis

All data are presented as the means ± SDs. All statistical data were analyzed by GraphPad Prism 7.0 (GraphPad Software). Statistical comparisons were measured by Student's *t*‐test or two‐way ANOVA with the Bonferroni multiple comparison test, and comparisons among more than three groups were measured by one‐way ANOVA with Dunnett's multiple comparison test. Kaplan–Meier analysis was performed using the log‐rank test. Statistical significance was designated with asterisks as follows: *, **, and *** indicate *p* < 0.05, *p* < 0.01, and *p* < 0.001, respectively.

## 3 RESULTS

### 3.1 Processing the data from the TCGA‐COAD database

**FIGURE 1:**
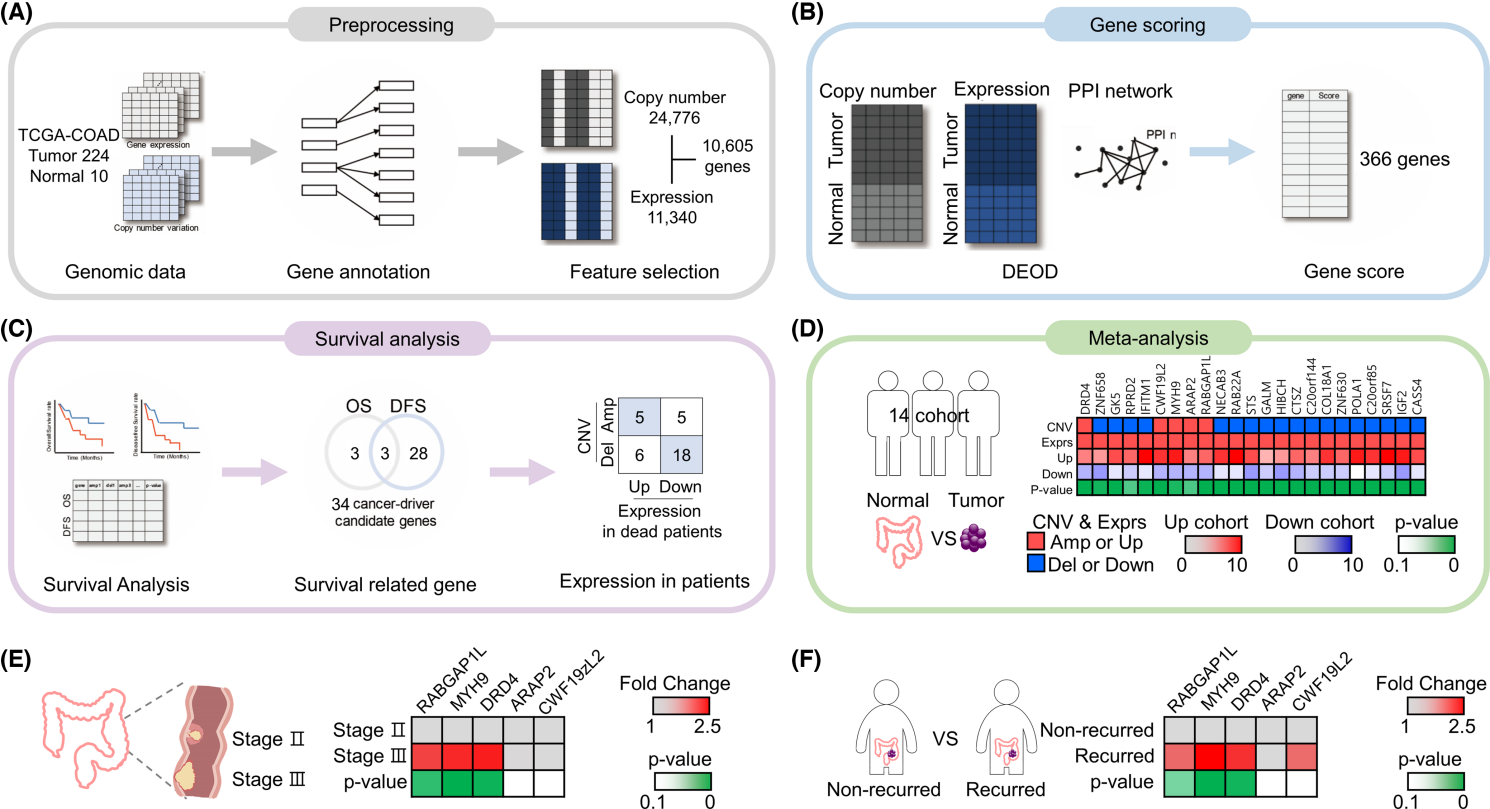
A schematic flow chart of our approach and identification of candidate genes. (A) The pipeline started with data collection and preprocessing from the TCGA‐COAD project, which consists of expression and CNV data. (B) The preprocessed data were sent to a gene scoring approach to compute weights for all effects of genes. (C) Survival analyses identified significant associations between the clinical prognosis of patients and CNV and expression. (D–F) We downloaded the expression data of each gene from public data sets. (D) Prognosis‐related genes were analyzed by meta‐analysis to identify a positive linear correlation between CNV and expression data in 14 data sets, which were the Alon,^17^ Gaedcke,^18^ Gaspar,^19^ Graudens,^20^ Kaiser,^21^ Ki,^22^ Kurashina,^23^ Notterman,^24^ Sabates‐Bellver,^25^ Skrzypczak,^26^ Skrzypczak2^26^ and Zou^27^ studies and the TCGA^28^ and TCGA2^28^ data sets provided by Oncomine. Then, we identified *RABGAP1L*, *MYH9*, *DRD4*, *ARAP2*, and *CWF19L2*. (E and F) We analyzed the five genes using the R2 platform to compare expression in stage 2 colorectal cancer (CRC) versus stage 3 CRC (GSE75316 for *RABGAP1L*, GSE37892 for *MYH9*, *DRD4*, *ARAP2*, and *CWF19L2*) and recurrent versus recurrent‐free tumors (xin130617 for *RABGAP1L*, GSE24551 for *DRD4*, and GSE18088 for *MYH9*, *ARAP2*, and *CWF19L2*).

CNV and gene expression data for 224 tumors and 10 normal samples were downloaded from the colon adenocarcinoma (COAD) project of The Cancer Genome Atlas (TCGA) database (Table 1).^16^ In total, 24,776 genes with log2 thresholds > 0.1 for amplification or lower than −0.1 for deletion were identified in tumor samples by using the Genomic Identification of Significant Targets in Cancer (GISTIC) 2 algorithm.^12^ Preprocessing of gene expression using DESeq2^13^ identified 11,340 protein‐coding genes that were differentially expressed in tumor tissues and normal tissues (*p* < 0.05). A comparison of the 24,776 genes identified from CNV preprocessing and the 11,340 genes identified from gene expression preprocessing revealed 10,605 overlapping genes (Figure 1A). To identify the cancer driver candidate genes that were pathogenic biomarkers, 10,605 genes were analyzed by the DEOD approach,^14^ and 366 genes were identified as cancer driver candidate genes (Figure 1B, Table S5 for detailed scores). These 366 genes were further screened to identify survival‐related genes in subsequent analyses.

**TABLE 1:** Clinical prognostic information on TCGA‐COAD patients

Category	Number of patients
Alive	198 (88%)
Deceased	26 (12%)
Disease free	163 (73%)
Recurrence	63 (27%)
Total	224

### 3.2 Association of molecular features with clinical prognosis

The threshold values shown in Table 2 were used to identify survival‐related cancer driver genes when grouping patients by the CNV values of the 366 genes identified by DEOD based on 224 tumor samples. For each of the 1%, 3%, and 5% thresholds, samples with values above the amplified threshold for each gene are classified as 1%, 3%, and 5% amplification groups of the gene, and samples below the deleted threshold are classified as 1%, 3%, and 5% del groups of the gene. Threshold values were determined as upper/lower values of 1%, 3%, and 5% of the total copy number. Excluding genes with no sample in either the amplification or deletion group, the numbers of genes in the 1%, 3%, and 5% groups that complied with the threshold values were 61, 212, and 302, respectively, for OS and 109, 247, and 323, respectively, for DFS (Table 3). Conducting survival analysis by plotting Kaplan–Meier curves for OS and DFS using these genes revealed that for OS, there were one, three, and four genes with significant differences between their amplification and deletion groups for the 1%, 3%, and 5% groups, respectively. For DFS, there were 5, 20, and 23 genes with significant differences between these groups (see Table S6 for the gene list). There were six genes that showed significant differences between the amplification and deletion groups for at least one threshold regarding OS; there were 31 corresponding genes related to DFS (Table S7). Among genes in the 1%, 3%, and 5% groups, a total of 34 genes, including three overlapping genes (*ARAP2*, *GK5*, and *RPRD2*), had significant effects on OS and DFS. Furthermore, these genes were compared with CNV data to identify relationships between the amplification of CNVs in tumors and their high expression in the cancer tissue samples of deceased patients compared with alive patients, or vice versa. Twenty‐three candidate genes were identified by survival analysis based on the TCGA‐COAD database (Figure 1C).

**TABLE 2:** Amplified and deleted group for each threshold

	CNV	Threshold		
		1%	3%	5%
Threshold	Amp	2.71	1.89	1.55
	Del	−2.95	−1.95	−1.52
Average *N* samples	Amp	2.4	5.7	9.7
	Del	2.4	5.5	8.9

*Note*: After listing all CN values in the data set, the upper and lower 1, 3, and 5% values were set as threshold values for grouping.

Abbreviations: Amp, amplified group; Del, deleted group.

**TABLE 3:** Number of genes for survival analysis by threshold

	Survival	Threshold		
		1%	3%	5%
Analyzed genes	OS	61	212	302
	DFS	109	247	323
Significant genes	OS	1	3	4
	DFS	5	20	23

*Note*: Analyzed genes, the number of genes for which survival analysis is possible among all candidate genes; Significant genes, the number of genes for which the results of survival analysis were significant.

Abbreviations: DFS, 5‐year disease‐free survival; OS, 5‐year overall survival.

### 3.3 Verification of candidate genes by in silico system analysis

We conducted verification of 23 candidate genes using the Oncomine and R2 platforms and in silico system analysis to evaluate markers to be used in the survival prediction model. CNV involves the amplification or deletion of 1 kb or larger DNA segments and promotes tumor progression via alteration of the expression levels of genes. Several studies have shown that amplification increases gene expression and deletion decreases gene expression, showing that CNV alterations are positively and linearly related to gene expression.^29^, ^30^, ^31^ Therefore, to match the CNV data with gene expression data, we collected the expression data of 23 genes in tumor tissues and normal tissues using 14 datasets from the Oncomine database and performed a meta‐analysis. We identified five candidate genes with higher gene expression associated with amplification in tumor tissues versus normal tissues: Rab GTPase activating protein 1‐like (RABGAP1L), myosin heavy chain 9 (MYH9), dopamine receptor D4 (DRD4), ArfGAP with RhoGAP domain ankyrin repeat and PH domain 2 (ARAP2), and CWF19‐like protein 2 (CWF19L2). There were no genes that had both lower gene expression and gene deletion in tumors compared with normal tissues (Figure 1D, Table S8).

To examine whether the five candidate genes were related to survival and recurrence, we conducted further analysis by exploring the RNA‐sequencing data of CRC patients using a public database, R2: Genomics Analysis and Visualization Platform (http://r2.amc.nl). Genes with high expression in advanced and metastatic cancer are associated with poor patient prognosis.^32^ Therefore, we compared the gene expression between nonmetastatic stage II CRC and metastatic stage III CRC. *RABGAP1L*, *MYH9*, and *DRD4* were significantly more highly expressed in stage III CRC than in stage II CRC (Figure 1E). Next, we verified the candidate genes by comparing the expression in recurrent tumors and nonrecurrent tumors. *RABGAP1L*, *MYH9*, and *DRD4* expression were significantly increased in recurrent tumors (Figure 1F). The results from the R2 platform indicated that the three genes affect the progression and recurrence of CRC and even the survival of CRC patients. Therefore, we identified three genes, *RABGAP1L*, *MYH9*, and *DRD4*, that may be used as biomarkers for survival prediction models.

### 3.4 Development of ML models to predict CRC patient survival

To predict the OS and DFS of patients with CC, we developed ML models using tumor stage features and the expression of three genes. There were some clinical features for predicting the survival of patients, such as age, tumor stage, and sex. In several studies, tumor stage features were the most significant for predicting survival.^8^, ^33^, ^34^, ^35^ Therefore, we selected tumor stage as a clinical feature to develop the ML models. Based on tumor stage features and the expression of the three identified candidate genes, *RABGAP1L*, *MYH9*, and *DRD4*, we developed logistic regression models for the prediction of CRC patient prognosis by ML. The baseline model is a logistic regression model using only AJCC tumor stage as a variable, and models 1–7 are logistic regression models using all possible combinations of three candidate genes and tumor stage as variables.

**FIGURE 2:**
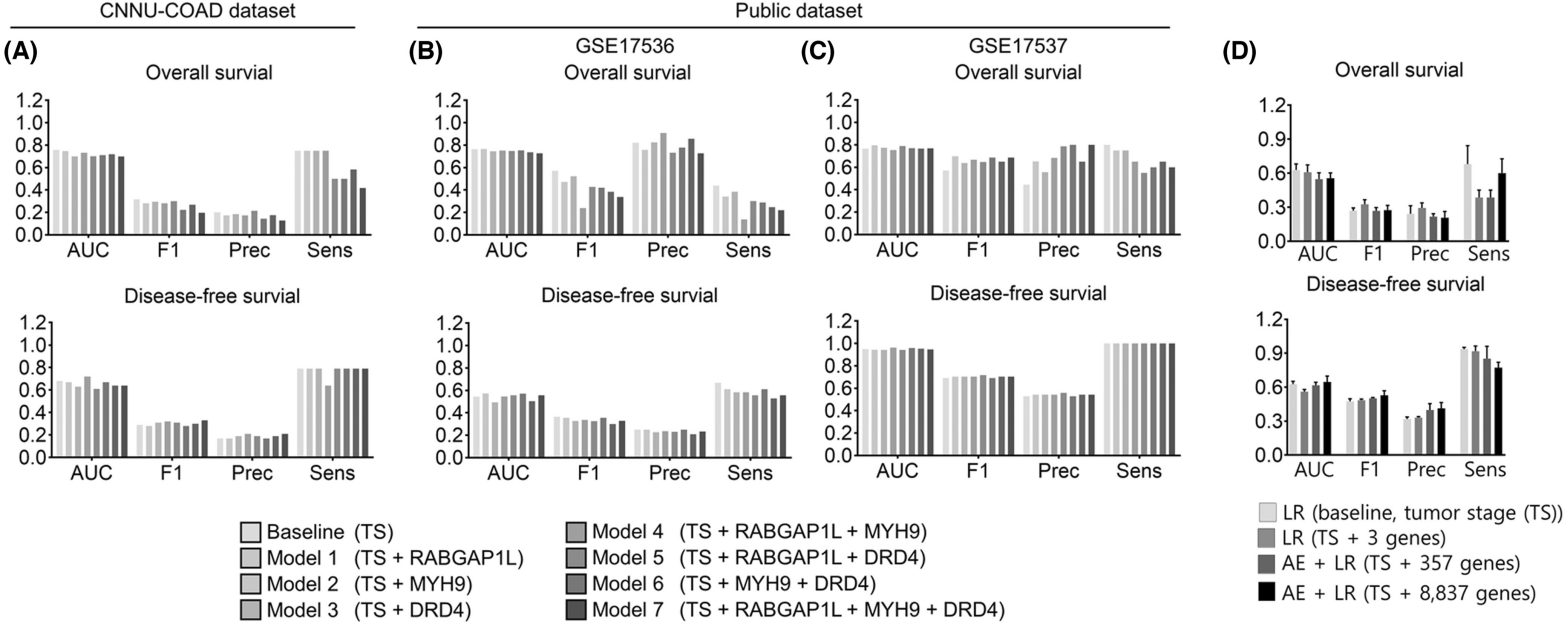
Survival prediction models. (A–C) AUC, area under the curve; F1, F1 score; Prec, precision; Sens, sensitivity. (A) To develop the survival prediction model, we trained the logistic regression model using the TCGA‐COAD data set to obtain tumor stage information and expression data for the three genes. We applied the survival prediction model to predict survival in the Chonnam‐COAD data set. (B, C) Prediction results of three public CRC patient cohorts: (B) GSE17536 and (C) GSE17537. Logistic regression models were trained with TCGA‐COAD and tested with each public cohort. (D) Each model is a logistic regression (LR) model. We used only tumor stage (TS) as the variable (baseline) or TS and three selected genes as variables. The autoencoder‐LR joint model used TS and 366 DEOD score genes as variables (AE + LR) and the AE + LR model used TS and 10,605 DEOD input genes as variables. All models were trained and tested with five‐fold stratified cross‐validation on gene expression data from 223 TCGA‐COAD patients. In all panels, data are reported as the means ± SEMs. AUC, five‐fold average area under the curve; F1, 5‐fold average F1 score; Prec, five‐fold average precision; Sens, 5‐fold average sensitivity

First, we trained the models using the gene expression profiles of the three genes and tumor stages from the TCGA‐COAD data set. To test which combinations of three gene expressions and tumor stage information are the best‐selected features for the prediction, seven trained models were tested using the Chonnam‐COAD data set (137 patients) (Figure 2B). The area under the ROC curve (AUC), F1 score, precision (Prec), and sensitivity (Sens) were used to evaluate the prediction performance. Interestingly, Model 1 (the model of tumor stage and *RABGAP1L*) showed the highest performance of OS predictions (AUC = 0.69, F1 score = 0.26) compared with baseline (AUC = 0.66, F1 score = 0.24) and other models (average AUC = 0.64, F1 score = 0.242) in Chonnam‐COAD. For the prediction of Chonnam‐COAD DFS prognosis, Model 3 (tumor stage + *DRD4*) had the highest AUC (0.72 vs. 0.64 ± 0.02), whereas Model 7 (tumor stage + *RABGAP1L* + *MYH9* + *DRD4*) had the highest F1 score (0.33 vs. 0.3 ± 0.015). In general, the prediction performance of the prediction models (Models 1–7) was better than that of the baseline variable (tumor stage) (Figure 2B, Table S9).

Additionally, seven trained models have tested in two public data sets from western populations (GSE17536 and GSE17537), which might show better performances as our gene selections were based on western cohorts (i.e., TCGA). When using GSE17536, Model 1 showed the highest AUC performance in OS and DFS (AUC of OS = 0.765, AUC of DFS = 0.572) compared with baseline (AUC of OS = 0.763, AUC of DFS = 0.544) and other models (average AUC of OS = 0.743; average AUC of DFS = 0.537) (Figure 2B, Table S10). In GSE17537, for the prediction of OS prognosis, Model 1 (AUC of OS = 0.796) and Model 4 (AUC of OS = 0.790) showed significantly higher prognostic predictive performance than baseline (AUC of OS = 0.766). For the prediction of DFS prognosis, Model 3 showed the highest AUC performance (AUC of DFS = 0.963) compared with baseline (AUC of DFS = 0.949) (Figure 2C, Table S11). In the Chonnam‐COAD data set and two public data sets, predictive models using the candidate genes as variables performed better than the baseline, especially in the case of analyzing public data sets, and most models had a high AUC value above 0.7. These results suggested that the candidate three genes had stable and robust predictive power as variables in multiple independent cohorts.

Next, we built two other predictive models: the first used candidate driver genes identified by DEOD, and the second used all the genes used as the DEOD input (Figure 2D). The first model used the expression values of 357 genes among 366 candidate genes, where genes with zero expression values were excluded. We also used an autoencoder model for dimension reduction, where hidden layers of 100, 3, and 100 dimensions were connected. The second model used the expression profiles of 8837 genes among 10,605 DEOD input genes after removing genes with zero expression values and low variations (< 10%). Based on the five‐fold cross‐validation of TCGA‐COAD data, we compared the performance of these two models with that of the logistic regression model using the three genes identified in this study. In OS prediction, the proposed logistic regression model using the three identified genes showed the highest F1 score (0.33 ± 0.09) and the second‐best AUC value (0.61 ± 0.15). In DFS prediction, although the proposed model had a lower F1 score (0.49 ± 0.03) than those of the other two models (0.50 ± 0.01 and 0.53 ± 0.09, respectively), it had a higher F1 score than the baseline (0.47 ± 0.05) using only the tumor stage as a variable (Figure 2D). In conclusion, the model with information on the expression of the three genes and tumor stage performed better than the model with only tumor stage and showed high performance because there was no significant difference in the performance of the model with a large number of genes.

### 3.5 Verifying the protumor activity of the three candidate genes

**FIGURE 3:**
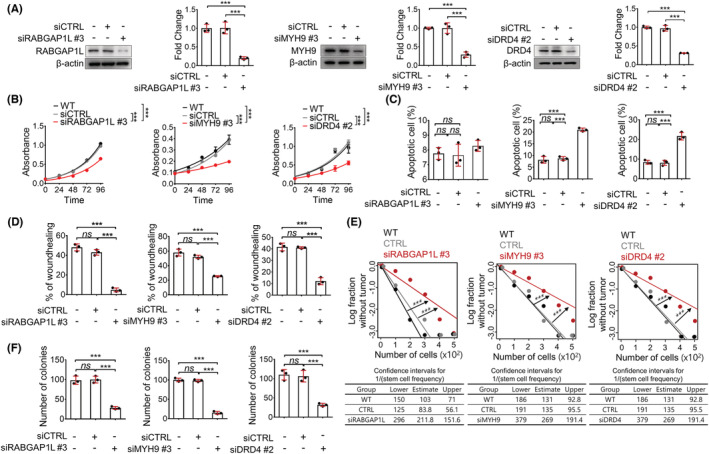
Verification of the protumor activity of the three genes. (A) Western blot assays confirmed the knockdown of the three genes. (B) MTT assays were performed to assess cell proliferation. The absorbance (570 nm) was measured at each time point (*n* = 5/each point). (C) Apoptosis assays were performed to evaluate the effect of the knockdown of the three genes on apoptosis. The percentage of apoptotic cells was analyzed by flow cytometry after staining the cells using Annexin V‐FITC and PI (*n* = 3/group). (D) Cell migration was assessed by the wound‐healing assay to compare the knockdown group with the wild‐type or control group. The wound area was photographed with a microscope at each time point (*n* = 3/group). (E) Limiting dilution assays were performed to assess the effect of the knockdown of the three genes on tumor initiation ability. Cancer cells at different dilutions were cultured in poly‐HEMA‐coated plates (*n* = 12/group). After 14 days, the number of wells with spheres was counted and analyzed by the extreme limiting dilution assay web tool. (F) Clonogenic assays were performed to assess the survival potential after the knockdown of the three genes. The number of colonies was counted after staining with crystal violet (*n* = 3/group). In all panels, data are reported as the means ± SEMs; *, **, and *** indicate *p* < 0.05, <0.01, and <0.001, respectively. Statistical comparisons between two groups were performed using Student's *t*‐test or two‐way ANOVA with the Bonferroni multiple comparison test or one‐way ANOVA with Dunnett's multiple comparison tests for three or more groups.

Because of the insufficient information on the role and function of the genes from computational analysis, there are premature or inappropriate uses of computational data before genes have been adequately tested and validated.^36^, ^37^, ^38^ Therefore, there is uncertainty regarding using these three candidate genes as therapeutic biomarkers. Therefore, to validate the computational data and evaluate whether the three genes contribute to malignant behavior in CC, we investigated the protumor activity by experimental analysis. Primarily, to perform the series of analyses, we determined the silencing effects of three different siRNA sequences targeting each gene and chose the siRNA that had the most potent silencing effect (Figure S2A) and confirmed the knockdown efficiency by checking the protein level (Figure 3A). Using siRNA for each of the genes, we first evaluated whether the three genes affect cell proliferation and apoptosis. Knockdown of each gene attenuated cell proliferation (Figure 3B). In apoptosis analysis, the knockdown of the *MYH9* and *DRD4* genes increased the number of apoptotic cells, except for the *RABGAP1L* gene (Figure 3C, Figure S2B). Second, the migration ability of the three genes was assessed by a wound healing assay. Knocking down each gene in all groups reduced the migratory ability (Figure 3D, Figure S2C). Therefore, all three genes regulated cell proliferation and migration, but only *MYH9‐* and *DRD4‐*regulated apoptosis.

Next, we investigated whether the three genes affected tumor‐initiating ability and cell survival. We conducted a limiting dilution assay to assess tumor‐initiating ability. The results revealed that silencing each gene impaired tumor‐initiating ability, as the frequency of sphere cells was significantly decreased (Figure 3E). Additionally, the knockdown group of each gene reduced the colony‐forming ability compared with the wild‐type group or the control group, revealing that all three genes affected cell survival ability (Figure 3F, Figure S2D). Inhibition of the expression of each gene in CC decreased the survival of tumor‐initiating cells. Thus, all three genes play a role in cell survival and tumor initiation.

Collectively, a series of analyses revealed the protumor activity of the three genes, *RABGAP1L*, *MYH9*, and *DRD4*. These results confirmed that these genes could be biomarkers for disease severity and therapeutic targets.

### 3.6 Prognostic value of the three genes used in survival prediction models

**FIGURE 4:**
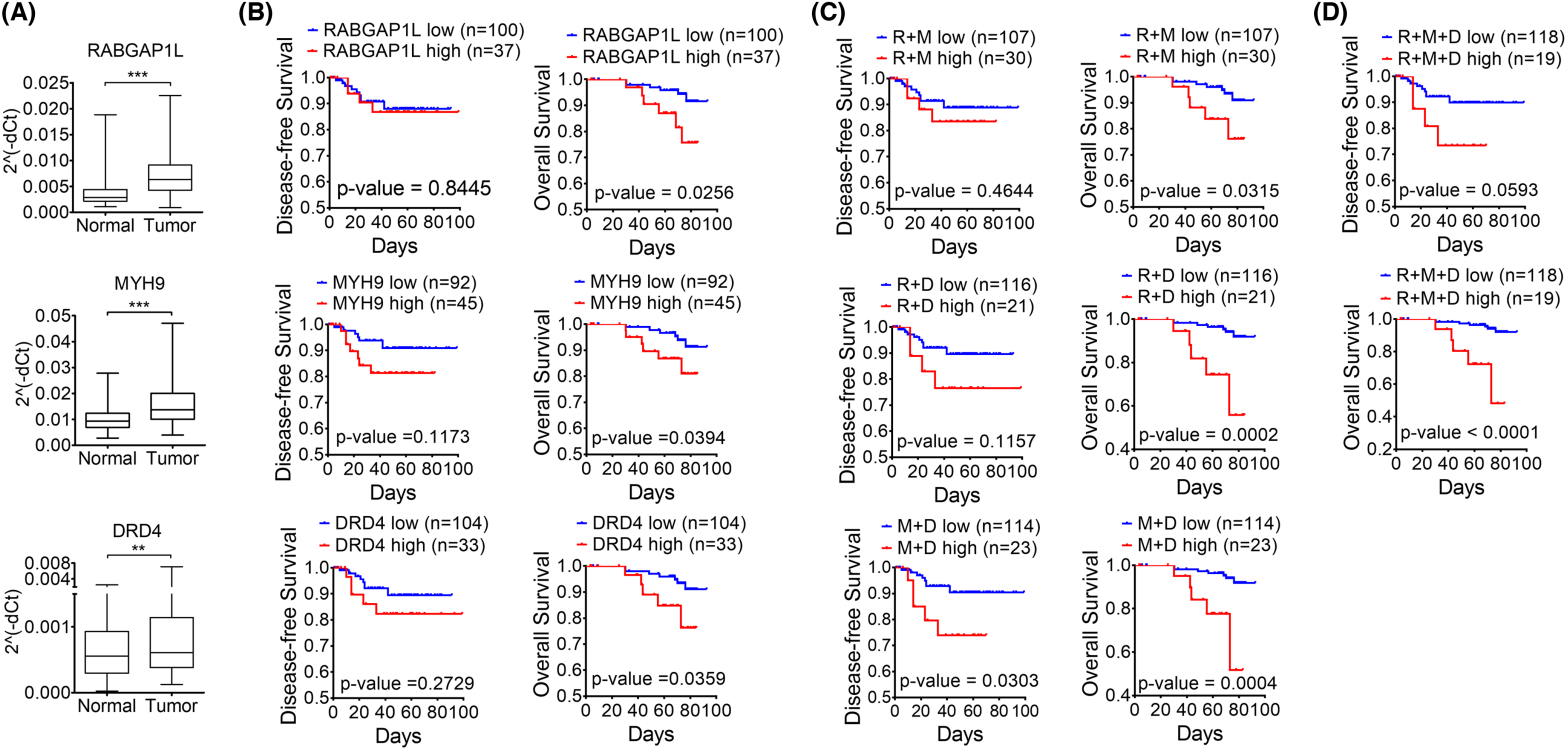
Correlation of the expression of the three genes in patient tissues and clinical outcomes. (A) The expression of each gene at the mRNA level was measured in CC patient‐derived tumor tissues and normal tissues from Chonnam National University Hwasun Hospital. (B‐D) Prognostic implications of multiple combinations of the three genes in patients with CC. Kaplan–Meier survival curve analysis was performed using data from 137 patients for disease‐free survival analysis and overall survival analysis. Patients were grouped based on the expression of a single gene (B), the coexpression of two genes (C), and the coexpression of all three genes (D). Statistical significance was determined by the log‐rank test. R: RABGAP1L, M: MYH9, D: DRD4

To evaluate the prognostic value of the three genes and the theranostic application of survival prediction models, clinical analysis was performed using CC patient data from Chonnam National University Hwasun Hospital. First, the expression of the three genes was higher in tumor tissue than in normal tissue of CC patients (Figure 4A). Next, we conducted DFS and OS analyses with multiple combinations of the three genes. When grouping by single genes, CC patients in the high expression group presented remarkably shorter OS than those in the low expression group, but in the DFS analysis, there was no significant difference between the high and low expression groups (Figure 4B). When the analysis was performed with two‐gene combinations of the three genes, the results indicated that only the group with high expression of both *MYH9* and *DRD4* presented significantly poorer DFS and OS than the groups with low expression. However, both groups that highly coexpressed *RABGAP1L* and *MYH9* and coexpressed *RABGAP1L* and *DRD4* showed poorer OS without altering DFS (Figure 4C). Finally, high expression of all three genes was significantly associated with a poor clinical outcome in terms of OS but not DFS (Figure 4D). According to the results, patients with high levels of both *MYH9* and *DRD4* had a lower DFS and OS and an adverse prognosis. Other combinations yielded a significant difference in only OS. Clinical validation suggested that a combination of the *MYH9* and *DRD4* genes is a prognostic biomarker that can provide insight into the survival prediction of CC patients.

## 4 DISCUSSION

This study identified survival‐related genes by integrating CNV and gene expression data using ML and further validated the genes using in silico system analysis, resulting in the discovery of *RABGAP1L*, *MYH9*, and *DRD4*. Using these three genes, we developed survival prediction models and confirmed their accuracy and performance in predicting CC prognosis by utilizing statistical estimation indicators. Moreover, the genes from the ML analysis were validated as potential therapeutic targets by experimental analysis and as diagnostic markers by clinical analysis. Thus, our survival prediction approach using ML, in silico system analysis and validation could be applied to patients.

To develop survival prediction models, previous studies revealed survival‐related genes by Bayesian network‐based methods that analyze diverse data types, such as copy number and gene expression data.^39^, ^40^ However, Bayesian network‐based methods do not fully integrate copy number and gene expression data.^41^, ^42^ In this study, we used an improved statistical approach, DEOD, which identified cancer driver genes by integrating diverse data types, such as CNV, mutations, and expression data, analyzing genetic alterations and directional relationships between genes across various data types.^14^ Cancer driver genes are pathogenic genes related to patient prognosis. Therefore, finding cancer driver genes by DEOD could pave the way for the identification of survival‐related genes in cancer patients. Additional verification was performed using in silico system analysis, such as the Oncomine and R2 platforms, which were constructed based on patients' genetic information. To identify the genes that are most suitable for predicting patient survival, we used the data from Oncomine to identify the genes whose CNV status and expression levels showed positive linear correlations, and the R2 platform was used to discover the genes that were related to cancer progression and recurrence. Through further verification of the data from the ML analysis by in silico system analysis, we identified *RABGAP1L*, *MYH9*, and *DRD4*, which could improve the performance of patient survival prediction. The survival prediction models were constructed using diverse combinations of these genes and applied to the in‐house cohort. Notably, the survival prediction models predicted the survival of CC patients with higher performance than the model based on only tumor stage information.

Prognostic values for various combinations of the three genes used to develop the survival prediction models were measured. The clinical analysis revealed that the coexpression of *MYH9* and *DRD4* was associated with significantly different survival rates between the high expression group and the low expression group in both the DFS and OS analyses. In parallel, a series of experimental analyses showed that *MYH9* and *DRD4* contributed to malignant tumor behavior, and these results suggested that *MYH9* and *DRD4* have potential as therapeutic targets in CC. Indeed, numerous studies have provided evidence of the oncogenic roles of *MYH9* and *DRD4* and suggested these two genes as potential therapeutic targets in diverse cancers. *MYH9* plays oncogenic roles in hepatocellular carcinoma by enhancing cancer stemness properties, metastasis, and proliferation.^43^, ^44^ *DRD4* plays a role in tumorigenicity and metastasis of glioblastoma.^45^ These previous studies firmly support our findings. Accordingly, in CC, further studies to understand the molecular mechanisms of *MYH9* and *DRD4* are expected to contribute to the proposal of new CC therapy strategies.

In summary, we developed a survival prediction model based on genes that were identified by ML and in silico system analysis. Furthermore, we verified genes that were used in the survival prediction model as diagnostic markers by clinical analysis and potential therapeutic targets by experimental validation. Our study provides survival‐related biomarkers and insight into the development of survival prediction models for CC patient survival to improve the performance of predictive approaches.

## 5 CONCLUSION

In this study, we identified genes related to CC survival and developed survival prediction models. We performed ML to screen survival‐related driver genes. Furthermore, in silico system analysis was performed to clinically assess data from the ML analysis, and we then identified *RABGAP1L*, *MYH9*, and *DRD4*. The survival prediction models based on the expression of these three genes and tumor stage information had higher performance than the model with only tumor stage information when applied to predict the prognosis of CC patients. Of note, among these three genes, the combination of *MYH9* and *DRD4* was verified to be a diagnostic biomarker by clinical analysis and a therapeutic biomarker with protumor activity by experimental analysis. Therefore, our survival predictive approach developed by ML, in silico system analysis and validation will provide information regarding CC patient survival and guide treatment decision‐making.

## AUTHOR CONTRIBUTIONS

**Choong‐jae Lee:** Data curation (lead); investigation (lead); methodology (lead); validation (lead); visualization (lead); writing – original draft (lead). **Bin Baek:** Data curation (lead); investigation (lead); methodology (lead); validation (lead); writing – original draft (lead). **Sang‐Hee Cho:** Funding acquisition (equal); project administration (equal); resources (lead). **Tae‐Young Jang:** Investigation (supporting); methodology (supporting); visualization (supporting). **So‐El Jeon:** Investigation (supporting); methodology (supporting); visualization (supporting). **Sunjae Lee:** Writing – review and editing (supporting). **Hyunju Lee:** Conceptualization (lead); funding acquisition (lead); project administration (lead); resources (lead); supervision (lead); writing – review and editing (lead). **Jeong‐Seok Nam:** Conceptualization (lead); investigation (lead); project administration (lead); resources (lead); supervision (lead); writing – review and editing (lead).

## FUNDING INFORMATION

This work was supported by the GIST Research Institute GIST‐CNUH Research Collaboration grant funded by the Gwangju Institute of Science and Technology (GIST) in 2021 and by “GIST Research Institure(GRI) IIBR” grant funded by GIST in 2022. Additionally, this work was supported by the National Research Foundation of Korea through a grant funded by the Korean government (Ministry of Science, ICT and Future Planning; NRF‐2020R1A2B5B03094382) and by a grant from the Cell Logistics Research Center of the National Research Foundation of Korea (NRF‐2016R1A5A1007318).

## CONFLICT OF INTEREST

The authors declare no conflict of interest.

## ETHICS APPROVAL AND CONSENT TO PARTICIPATE

The biospecimens of normal and tumor tissue from each patient were provided by the Biobank of Chonnam National University Hwasun Hospital, a member of the Korea Biobank Network, with informed consent. This study was approved by the Chonnam National University Hwasun Hospital Institutional Review Board (approval number: IRB CNUHH‐2020‐173) and undertaken in accordance with the Declaration of Helsinki.

## Supporting information


Appendix S1

## Data Availability

The data sets and methods used and/or analyzed in the current study are available within the manuscript or its supplementary information files. All data analyzed and materials used in this study are available from the corresponding author upon reasonable request.
