# Supplementary material for: Machine learning with in silico analysis markedly improves survival prediction modeling in colon cancer patients
Source: Cancer Med. 2022 Nov 7;12(6):7603–15. doi: 10.1002/cam4.5420 (PMC10067044; doi:10.1002/cam4.5420)
Supplement: Supplementary file 1 — Appendix S1 [file CAM4-12-7603-s001.pdf]

Supplementary Materials for

Machine learning with *in silico* analysis markedly improves survival prediction modeling for colon cancer patients

Choong-Jae Lee[1]†, Bin Baek[2]†, Sang Hee Cho[3]†, Tae-Young Jang[1], So-El Jeon[1], Sunjae Lee[1], Hyunju Lee[2]*, Jeong-Seok Nam[1,4,*]

[1] School of Life Sciences, Gwangju Institute of Science and Technology, Gwangju, 61005, Korea

[2] School of Electrical Engineering and Computer Science, Gwangju Institute of Science and Technology, Gwangju, 61005, Korea

[3] Department of Hemato-Oncology, Chonnam National University Medical School, Gwangju, 61186, Korea

[4] Cell Logistics Research Center, Gwangju Institute of Science and Technology, Gwangju, 61005, Korea

[*] Correspondence: namje@gist.ac.kr (J.-S.N.), hyunjulee@gist.ac.kr (H.L.); Tel.: +82-62-715-2893 (J.-S.N.), +82-62-715-2213 (H.L.)

†These authors contributed equally to this work.

1. Supplementary Materials and Methods

1.1 ML-based Survival Analysis

To determine how the copy number variations (CNVs) or expression of the candidate cancer driver genes affected the clinical prognosis of patients with colorectal cancer (CRC), Kaplan–Meier survival curves were plotted for overall survival (OS) and disease-free survival (DFS) in each of the amplification and deletion groups. The cutoffs for identifying copy number changes in each sample can differ depending on the data [1, 2]. We selected the cutoffs of 1%, 3%, and 5% used in a previous study [1] for determination of amplifications and deletions. For each candidate cancer driver gene, patients with extreme amplification (those with CNV segment values in the top 1%, 3%, and 5%) were labeled the amp groups, while patients with extreme deletion (those with CNV segment values in the bottom 1%, 3%, and 5%) were labeled the del groups. Patients with CNV values within 1%, 3%, and 5% of the upper and lower groups were included in each group, and a survival analysis was performed between the two groups. The *Survfit* function of the "survival" package (v.3.2.7) in R was used for this, while the *Survdiff* function in the same package was used for the log-rank test.

1.2 RNA Isolation and Real Time-Quantitative Polymerase Chain Reaction (RT–qPCR)

For RNA isolation from the cell line, total RNA was extracted using RNAiso (Takara, Shiga, Japan), and RNA concentration and purity were measured using a nanodrop instrument (Thermo Fisher Scientific, Waltham, MA, USA). The RNA purity level was analyzed using the 260/280 absorbance ratio. RNA was converted into cDNA by using the PrimeScript 1st strand cDNA Synthesis Kit (Takara), and 600 ng of the cDNA was subjected to PCR using Power SYBR Green PCR Master Mix (Applied Biosystems, Foster City, CA, USA). Real-time qPCR was carried out using a StepOnePlus Real-Time PCR System (Applied Biosystems). The relative mRNA expression of selected genes was normalized to peptidylprolyl isomerase A (PPIA) and quantified using the $\Delta\Delta$Ct method.

1.3 Protein Isolation and Western Blotting

Cells were lysed using RIPA buffer (20 mmol/l Tris-HCl, pH 7.5, 200 mmol/l NaCl, 1% Triton X-100, 1 mmol/l dithiothreitol) containing protease inhibitor cocktail (Roche, Basel, Switzerland). Protein concentrations were measured with a BCA assay kit (Thermo Fisher Scientific), and 10 μg of protein was loaded in an 8% SDS–PAGE gel, separated by SDS–PAGE and transferred to polyvinylidene difluoride membranes that were activated with methanol before use. BSA (10%, Bovogen Biologicals, Melbourne, Australia) was used to block the membrane. The membrane was then incubated with the appropriate primary antibody and horseradish peroxidase-conjugated secondary antibody. β-actin was used as a loading control. The antibodies used for the Western blot assay are listed in Table S3.

1.4 Cell Viability Assay

Cells were seeded at $5x10^3$ cells/well in 96-well plates and grown for 12, 24, 48, 72, and 96 h in serum-supplemented media. At 12, 24, 48, 72, and 96 h, cell viability was assessed by thiazolyl blue tetrazolium bromide (MTT, Sigma–Aldrich, St. Louis, MO, USA) according to the manufacturer's instructions. The relative cell viability was measured at a wavelength of 570 nm using an Epoch microplate reader (Biotek, Winooski, VT, USA).

1.5 Clonogenic Assay

After siRNA transfection, cells were plated in triplicate 12-well plates at 500 cells/well for 10 days at 37 °C in a humidified incubator with 5% CO_2. Then, the cells were stained with 0.1% crystal violet and dried overnight. Colonies stained with crystal violet were photographed (Nikon, Tokyo, Japan), and the number of colonies was counted using Image-Pro Premier 9.0 (Media Cybernetics, Rockville, MD, USA).

1.6 Apoptosis Assay

Quantitative analysis of the apoptotic cells was performed using the Annexin V-fluorescein isothiocyanate (FITC) Apoptosis Detection Kit I (BD Biosciences, San Jose, CA, USA). After siRNA transfection, the cells were collected and washed twice with cold PBS. The cells were resuspended in 100 μl of binding buffer ($1x10^5$ cells) and stained with 5 μl of FITC Annexin V and propidium iodide

(PI). Then, the mixture was incubated at room temperature for 15 min in the dark. After incubation, 400 μl of binding buffer was added, and the samples were then analyzed by flow cytometry (BD AccuriTM C6, BD Biosciences).

1.7 *In Vitro* Limiting Dilution Assay

To examine the tumor initiating ability, cells were seeded in sphere culture-conditioned 96-well plates (poly-HEMA-coated plates; poly 2-hydroxyethyl methacrylate, Sigma–Aldrich) at varying cell densities of 100, 200, 300, 400, and 500. After 14 days of culture, the wells with spheres were counted (n=10/group) and analyzed by the extreme limiting dilution assay webtool (http://bioinf.wehi.edu.au/software/elda).

1.8 Wound Healing Assay

Cells were seeded at $8x10^4$ cells/well in IBIDI medium (IBIDI, Lochhamer, Germany). After 24 h, the IBIDI medium was removed, and 1 mL of culture media with 10 nM cycloheximide (Sigma–Aldrich) was added. The wound area was photographed with a microscope (Am Leitz-Park, Wetzlar, Germany). The wound area was measured and recorded at 72 h and compared with the initial wound area at 0 h to determine the wound healing rate. The images were analyzed at each time point using ImagePro Premier 9 (Media Cybernetics).

1.9. Survival Analysis Using Patient Samples

Using data for the expression of each gene in patients, we divided patients into groups with high or low expression as previously described [3, 4]. Patients with gene expression higher than the average of all patients were defined as the high group, and patients with lower expression than the average were defined as the low group. When two or three genes were combined, patients whose expression of all the combined genes was higher than each average were defined as the high groups, and the other patients were defined as the low groups. After grouping, we analyzed the OS and DFS. Survival was calculated using the Kaplan–Meier method, and comparisons were performed using log-rank tests.

1.10. *In silico* system analysis.

We retrieved the complete datasets of CRC patients using oncomine, and thus we obtained expression data in CRC and normal colon tissues from 14 cohorts with transcriptome data including our candidate genes (Cohorts name: Alon [5], Gaedcke [6], Gaspar [7], Graudens [8], Kaiser [9], Ki [10], Kurashina [11], Notterman [12], Sabates Bellver [13], Skrzypczak [14], Skrzypczak2 [14], and Zou [15] studies and the TCGA [16] and TCGA2 [16] datasets). Based on expression data, we calculated the fold change in CRC tissue compared to normal colon tissue and performed a meta-analysis to combine the data from diverse datasets using METAL software [17]. As a result, the gene groups were divided into genes whose expression in CRC tissues increased or decreased significantly compared to normal (z-score > 2, p-value <0.05, Supplementary Table S8). Additionally, using R2, we analyzed differences in gene expression in each stage and differences in recurrent CRC compared with nonrecurrent CRC using 5 cohorts (GSE75316, GSE37892, xin130617, GSE24551, and GSE18088).

2. Supplementary Table

2.1 Table S1. Chonnam-COAD patient characteristics

**Characteristics**	**Number of patients**
Age, median (range)	70 (31-87)
Sex, n (%)	26 (12%)
Male	73 (53%)
Female	65 (47%)
AJCC stage	
II high risk	74 (54%)
III	64 (46%)
Alive	125 (91%)
Deceased	12 (9%)
Disease free	123 (90%)
Recurrence	14 (10%)
Total	137

2.2 Table S2. List of siRNA sequences

Target	Number	Sense	Antisense
**RABGAP1L**	#1	GCUAUGAUGGGAGAGCUUA	UAAGCUCUCCCAUCAUAGC
	#2	GAUUACAGUGCAGCAACUU	UGAAGGUAAACACAUCACU
	#3	AGUGAUGUGUUUACCUUCA	UGAAGGUAAACACAUCACU
**MYH9**	#1	AGAGUAGCUCGUCCUCACU	AGUGAGGACGAGCUACUCU
	#2	AGUAAGCUGGUUUACAGAU	AUCUGUAAACCAGCUUACU
	#3	GUGGUUUACCUGCACCGUU	AACGGUGCAGGUAAACCAC
**DRD4**	#1	CUGUCUUCAACGCCGAGUU	AACUCGGCGUUGAAGACAG
	#2	GCGCUUUUGUACGUUAAUU	AAUUAACGUACAAAAGCGC
	#3	GAGGGCGCUUUUGUACGUU	AACGUACAAAAGCGCCCUC

2.3 Table S3. List of primer sequences used for real-time PCR

	Primer		
**Target**	**Number**	**Sequence**	**Annealing Temperature**
**PPIA**	Forward<br>Reverse	TGCCATCGCCAAGGAGTAG<br>TGCACAGACGGTCACTCAAA	60 °C
**RABGAP1L**	Forward<br>Reverse	AGGTAACTCCAGCTGTTGCAT<br>GTCTTGGAACAGTTCACCTGC	60 °C
**MYH9**	Forward<br>Reverse	GGCACGGAAGGCTAAGCAAG<br>TGGTGACTTATAGCCAGGACC	60 °C
**DRD4**	Forward<br>Reverse	CAGACTCCACCGCAGACC<br>GTGACGGCGCTGACCA	55 °C
**RABGAP1L**	Forward<br>Reverse	GTCATCACTGGCATGTGGAACC<br>TCTCCAGGAGAAAGCGAACAGG	62 °C
**MYH9**	Forward<br>Reverse	ATCCTGGAGGACCAGAACTGCA<br>GGCGAGGCTCTTAGATTTCTCC	62 °C
**DRD4**	Forward<br>Reverse	CCTGCGGCTCCAACTGTGC<br>GGAAGGCCCCGACCACCAC	62 °C
**ARAP2**	Forward<br>Reverse	GTCTTCCCAGTCCACATTCCTC<br>GAAGCCTCCTTCCAAAACACACC	62 °C
**CWF19L2**	Forward<br>Reverse	GCTATTGCTGAGCATCGGAGTC<br>CCTCAGTAAGAGACCGTACGTTG	62 °C
**ACTB**	Forward<br>Reverse	AGCCTCGCCTTTGCCGA<br>CTGGTGCCTGGGGCG	62 °C

ACTB, β-actin

2.4 Table S4. List of antibodies used for Western blotting

Target	Origin	Conjugation	Corporation	Catalog #
**RABGAP1L**	Monoclonal Mouse	Unconjugated	Abnova	H00009910-M05
**MYH9**	Polyclonal Rabbit	Unconjugated	Invitrogen	PA5-17025
**DRD4**	Polyclonal Rabbit	Unconjugated	Solarbio	K007417P
**HRP Goat Anti-Mouse Ig**	Polyclonal Goat	Peroxidase-conjugated	BD Pharmingen™	554002
**HRP Goat Anti-Rabbit Ig**	Polyclonal Goat	Peroxidase-conjugated	BD Pharmingen™	554021

2.5 Table S5. Detailed score of cancer driver genes

Gene	Score	Gene	Score	Gene	Score
AHR	713.1548286	LPIN3	13.02920441	UNC5B	10.33310819
PREX1	660.9671845	ARHGEF9	12.99808646	ATP11A	10.31804612
GALR1	504.2246604	SNAI1	12.93395396	TSC22D3	10.30208938
CHD6	490.2994148	CA8	12.93170098	NOTCH1	10.29003516
LIME1	134.3893783	VMA21	12.76247531	AGL	10.28049977
ZNF407	113.5664039	CCDC68	12.75945821	HELZ2	10.2170186
PLCG1	110.4498885	ERBB3	12.75165249	PDE4D	10.20973587
SLC2A4RG	109.5769728	SGCD	12.70143046	COX10	10.18584642
FAM72D	75.22961885	PCDHA6	12.67859824	PCDH15	10.13789921
ATP9B	72.50753063	NRXN1	12.66786073	PRB1	10.13178029
POU5F1B	70.88665574	REM1	12.66659835	WISP2	10.11618156
PSD2	68.98718328	PAG1	12.6435199	MSLNL	10.10070115
KLF5	68.06728476	DRD4	12.63680519	C16orf70	10.0264496
RBL1	58.40781003	CYP2W1	12.62496037	NPIPB11	10.00967783
UGT2B4	55.90546882	RSRC1	12.54224123	PRIM2	10.00696225
ACTL10	55.67563878	ASB5	12.50087629	PSMA8	10.0047554
QKI	52.06872495	PEMT	12.49939244	TONSL	10.00472967
PTPN1	48.95136997	ST6GAL2	12.49323933	SMCO2	9.996321953
ZNF518A	48.68557866	COL10A1	12.4905863	WIPI1	9.984258491
CDH19	47.31961226	ATP2B3	12.48993265	DSC2	9.934415834
MRC1	43.65268299	CYSLTR1	12.47760364	ZP3	9.929357193
PRSS1	40.33913421	ANO2	12.47333281	PPM1F	9.902159445
GADL1	39.65236532	BIRC6	12.426974	MTG2	9.877836766
CCBE1	39.55270468	ARHGEF5	12.40924965	TNIP1	9.8706706
IFITM1	33.54502464	DENND3	12.39599629	RUNX1T1	9.825708231
RBFOX1	32.83776994	TYW1B	12.37140681	CTSZ	9.783177345
RNASEH2B	30.90474746	PLA2G4F	12.36893715	GALM	9.778977566
NEDD4L	30.8171728	CAMKK1	12.36070643	BORA	9.716798081
WWOX	30.34948867	POU2F2	12.24519423	MYO5B	9.714394018
ORM1	29.86361654	FAM117B	12.21509401	SH3BGRL	9.709438647
SLC26A10	27.86569193	GRP	12.21453876	FAM210B	9.692037743
SMAD4	27.74521787	CHST5	12.20539465	MTG1	9.685466979
TCF4	27.42789348	THOC1	12.12367665	PAPSS2	9.642285576
SYT10	27.36734471	DYNLRB1	12.10906851	CKMT1A	9.62581458
TPD52L2	27.08948601	PADI3	12.09916197	PLAGL2	9.603800804
ADNP	26.75067006	UPP2	12.06509759	SNRPN	9.581314163
GFPT1	26.49417002	KCNIP4	12.05605624	PIBF1	9.556316428
PTPRT	25.52730243	GMEB2	12.04649324	CD160	9.551860191
E2F1	25.24821579	ZNF658	12.0287499	SALL4	9.547956325
TNFRSF11B	25.2253076	CASP5	12.01922886	SPSB1	9.528096291
ADIPOR2	23.61022172	RRP7A	12.00371184	TTC27	9.502987289
PTPRM	22.76368836	ACKR2	12.00023043	ZNF626	9.484251517
EXT1	22.38303782	KRT23	11.86997632	C1QTNF9	9.47473381
CFH	21.90555266	RPA4	11.85896687	CELF4	9.462402892
LRWD1	21.89699104	GALNT13	11.82215887	HSBP1L1	9.450567986
B4GALT5	21.03401535	WSCD1	11.81329687	CTDSPL	9.40654196
DAPL1	20.64936898	MMP9	11.79951374	HBS1L	9.382993446
GPC4	20.44571426	GYPE	11.79141472	NCOA7	9.372540845
GPR89A	20.32271984	SRMS	11.74631927	CHD7	9.367726568
SLC14A2	20.20204145	GRHL1	11.71821232	RPRD1A	9.312210525
PRPS2	20.00601736	SLC17A9	11.69350869	AFF1	9.295239965
C20orf144	19.85680254	CTNNA3	11.66000644	SAMSN1	9.291343753
SPATA25	19.72578508	NAALADL2	11.63741972	SLITRK4	9.223883887
MRGPRF	19.49841074	ZNF630	11.61198557	RMND5A	9.212409471
SAMD10	19.31816172	EIF2S2	11.59610162	PPP2R2C	9.197963076
DPP6	18.72457374	NCOA5	11.59200405	MZT2A	9.173424259
LAMA5	18.29494105	CDRT1	11.56745451	TMEM204	9.130358364
ZNF385B	18.23735197	MEGF11	11.55150202	LIPA	9.128349542
CHCHD10	18.09718299	CREG2	11.52890751	MSC	9.079175137
AKAP14	17.55456059	ADAMTSL3	11.51918547	ZNF473	9.073228986
UCKL1	17.52527471	PRSS50	11.5016845	KIAA1328	9.034579827
CSMD1	17.41580762	NPHP4	11.48081533	BCAS4	9.02785636
CHODL	17.38556979	GAS7	11.4700681	ATP1B2	9.019299767
TLR8	17.19532369	TMEM201	11.46111346	FAM84B	9.009597323
AFF2	17.09393993	DOK5	11.44491811	RPL21	9.001841553
ZNF132	17.03840102	HIP1	11.42927657	PIP5K1B	8.987469487
CNGA3	16.94811834	TRPA1	11.42600489	DCDC2	8.977079381
SIGLEC5	16.87191716	ANKRD36C	11.42543694	RPRD2	8.958722471
SNTB1	16.86763463	HS6ST2	11.41173264	STS	8.927841339
COL18A1	16.65935956	RAVER2	11.31848028	HNRNPA1P48	8.919278217
PSAPL1	16.60378897	MBP	11.27895121	SORBS2	8.891163141
DOK6	16.5137743	ATP12A	11.27527192	MYH9	8.884203901
EMILIN3	16.46855261	GALNT15	11.26717486	CHRDL1	8.825171585
ADH1B	16.28636498	FRK	11.24203356	RPN1	8.744041304
TPX2	16.04071821	THRB	11.23612357	SLPI	8.648588038
ERG	16.0299746	ROCK1	11.21305385	BCL2L1	8.636346501
PTK2	15.9990373	HM13	11.2086062	FRMPD4	8.616771151
NPBWR1	15.99326494	AADAC	11.20651817	FCGR3A	8.587059734

WDR72	15.85628951	CASS4	11.17253709	DNAJC24	8.539999126
GEMIN6	15.57028025	MAPK4	11.15643388	ZHX1	8.449140828
GOLGA8B	15.56140684	FOXS1	11.13361945	C20orf85	8.445931169
PDZK1	15.44518116	WWC3	11.12546341	MTTP	8.410660904
TRIM16	15.41785767	GPR143	11.09050029	TMEM132B	8.373438447
KCNB2	15.36039906	CAD	11.06274108	IGF2	8.330518228
NECAB3	15.26311728	ARAP2	11.04995841	TSHZ1	8.300786587
TFEC	15.13789161	SRSF6	11.04966752	PCDH11X	8.245721939
SLC6A13	15.11318879	CELA3A	11.04401142	SLC24A4	8.214451091
SLC25A24	14.96636245	TXNL4A	11.01238038	MYH7B	8.098875765
WDR7	14.89106823	MGAM	11.00475549	KHDRBS2	8.025055632
LYST	14.76147982	EIF4EBP1	10.95130408	DENND4A	7.977514991
PRRG3	14.631444	HIBCH	10.90002605	ASXL1	7.963930569
EDN3	14.57839202	ZBTB7C	10.89545894	POFUT1	7.927857327
GK5	14.44321431	B3GALT5	10.81735503	RPL27A	7.84828008
EIF3C	14.37498211	LANCL3	10.78940775	RABGAP1L	7.757866929
ZDHHC15	14.34103388	MRGBP	10.7889379	ADRM1	7.737069456
COL14A1	14.31757613	ELAVL1	10.78759257	STOX2	7.707470753
BTNL3	14.30587935	NPIPA8	10.76444722	ZNF850	7.644035593
DSG3	14.25321455	CDC42EP3	10.75133201	POLR3C	7.617896158
MBD2	14.20183021	POLA1	10.73852132	CPSF1	7.566140487
CCL11	14.19214231	ECE1	10.62652216	ZMYM5	7.518742886
PADI1	14.18783893	FBXL7	10.62612826	CSE1L	7.412931873
TXK	14.03868602	MTSS1	10.60196012	BUD31	7.261823696
TPI1	13.99507965	IGFBP7	10.58980873	YTHDF1	6.786603819
RPS21	13.94657149	MAP1LC3A	10.57594733	CWF19L2	6.500018535
CTNNBL1	13.90316808	STK32B	10.57519193	RNF115	6.469317376
PDE9A	13.83766652	AUH	10.54529892	ZADH2	6.452206146
FAM19A5	13.83280971	AGR3	10.53076755	FBXO8	6.442919255
POLR2J3	13.80971947	GBP3	10.51383464	NKAP	6.370811144
KMT2E	13.76019257	JAK1	10.50962311	RAB22A	5.566082179
SLC32A1	13.66272816	ME2	10.49015974	NAP1L3	2.643944981
MYC	13.63449942	CDKN2A	10.48865037	FAM133A	2.363303953
SPIDR	13.53922508	ZNF570	10.45460065	ACSL4	2.019843688
KIF1B	13.43955324	STIM1	10.44606997	GPCPD1	1.765072618
LGALS9C	13.43605508	PHKG2	10.43308233	STMND1	1.6427745
TMEM189	13.34020679	NOTCH2NL	10.42842139	RPS6KA6	1.60462391
TAF4	13.29029073	PAK3	10.40030119	PRKCA	1.456444386
SULF1	13.26082689	ZNF217	10.39849463	FAM107B	1.418683543
APOBEC3A	13.25243918	DAP3	10.39548504	GNG3	1.385733462
AQP4	13.22693277	ACOT11	10.391241	DLEU7	1.26056939
SEMA3A	13.13201533	SRSF7	10.37856829	TAF1C	1.153803645
G2E3	13.11006512	TFAP2C	10.34644709	GYG2	1.121069252
CEBPB	13.03472535	SLC2A13	10.34137665	HNRNPUL2	1.106624893

2.6 Table S5. Gene list with significant difference in survival analysis

	Gene	CN	amp1	del1	amp1_event	del1_event	p_value1	amp3	del3	amp3_event	del3_event	p_value3	amp5	del5	amp5_event	del5_event	p_value5	pare with o	p-value1	p-value3	p-value5	mpare with	p-value	chment_pat	#BP	#CC	#MF	#KEGG	#TargetGene
	GK5	del	3	2	0	2	0.038947	8	2	1	2	0.0009	13	4	2	2	0.023745	down	0.233359152	0.233359152	0.233359152	up	0.360124093	0	0	0	0	0	64
	ARAP2	amp	1	3	1	0	0.157299	1	10	1	1	0.004678	5	18	1	2	0.499944	down	-	-	-	down	0.446296384	0	0	0	0	0	62
	RPRD2	del	NA	NA	NA	NA	NA	10	1	2	1	0.001565	19	6	2	1	0.075156	down	-	-	-	down	0.52833752	0	0	0	0	0	92
	DRD4	amp	1	2	1	0	1	3	5	2	2	0.062448	5	8	3	2	0.009391	up	-	0.064912332	0.289075155	down	1.94E-01	0	0	0	0	0	35
	IFITM1	del	2	3	1	1	0.317311	6	5	2	3	0.058782	8	6	2	3	0.033584	down	-	0.035907536	0.035907536	down	6.11731E-20	5	0	3	0	2	97
	ZNF658	del	NA	NA	NA	NA	NA	11	3	0	2	0.066821	19	7	0	3	0.003818	down	-	0.146649321	0.071342919	up	0.018627179	0	0	0	0	0	153
Survival	COL18A1	del	3	2	0	1	0.317311	7	12	0	3	0.273672	8	16	0	4	0.136707	down	-	0.381964124	0.963256921	down	2.76851E-05	138	87	29	17	5	503
	GOLGA8B	amp	NA	NA	NA	NA	NA	NA	NA	NA	NA	NA	NA	NA	NA	NA	NA	up	-	-	-	down	7.30E-07	0	0	0	0	0	292
	KIF1B	del	NA	NA	NA	NA	NA	1	14	0	2	0.256839	3	21	0	4	0.124058	down	-	1.55E-10	0.413097884	up	0.004087488	73	72	1	0	0	398
	NAALADL2	amp	1	2	0	0	-	4	10	0	0	-	6	14	0	0	-	down	-	-	-	down	5.75E-03	3	0	2	0	1	51
	NRXN1	amp	NA	NA	NA	NA	NA	3	15	0	1	1	9	16	1	1	0.220671	down	-	-	-	up	1.82E-02	0	0	0	0	0	109
	QKI	del	NA	NA	NA	NA	NA	2	8	0	1	0.59298	9	10	0	2	0.157299	down	-	-	0.706074066	up	0.139480465	9	9	0	0	0	140
	TMEM204	del	NA	NA	NA	NA	NA	4	1	0	0	-	9	2	0	0	-	up	-	-	-	down	0.007540592	0	0	0	0	0	24
	WDR72	amp	NA	NA	NA	NA	NA	NA	NA	NA	NA	NA	2	20	0	2	1	up	-	-	-	down	8.59E-23	44	26	15	3	0	99
	GK5	del	3	2	0	2	0.038947	8	2	3	2	0.0009	13	4	4	2	0.044096	down	0.233359152	0.233359152	0.233359152	up	0.360124093	0	0	0	0	0	64
	CASP5	amp	2	3	2	0	0.038947	2	7	2	3	0.002624	3	10	2	4	0.088593	down	0.10364303	0.10364303	0.10364303	up	0.000221927	0	0	0	0	0	56
	IGF2	del	7	1	3	1	0.014306	13	2	6	2	0.405784	15	6	7	2	0.759491	down	-	9.18642E-05	9.18642E-05	down	7.86E-05	0	0	0	0	0	52
	RBFOX1	amp	1	5	1	1	0.0455	3	11	1	4	0.716861	4	14	1	4	0.934633	down	-	-	-	up	1.18E-02	465	371	44	44	6	1234
	ZNF630	del	7	5	1	2	0.029336	9	8	2	3	0.10281	14	10	6	5	0.160952	down	0.600253576	0.376178879	0.058742638	down	1.78676E-06	77	52	18	7	0	112
	C20orf144	del	NA	NA	NA	NA	NA	4	1	1	1	0.0455	13	1	4	1	0.000911	down	-	-	-	down	4.54004E-18	448	358	39	23	28	804
	C20orf85	del	NA	NA	NA	NA	NA	5	1	1	1	0.025347	15	2	4	1	0.041908	down	-	-	-	down	1.23E-01	19	14	0	5	0	119
	CASS4	del	NA	NA	NA	NA	NA	5	1	0	1	0.025347	11	1	2	1	0.000911	down	-	-	-	up	0.006198877	0	0	0	0	0	186
	CTSZ	del	NA	NA	NA	NA	NA	4	1	1	1	0.0455	11	2	3	1	0.019016	down	-	-	-	up	0.002160123	0	0	0	0	0	40
	CWF19L2	amp	1	4	1	3	0.350816	2	11	2	4	0.010691	6	19	4	7	0.034032	up	-	0.214416101	0.014720942	down	2.80E-04	0	0	0	0	0	143
	DOK5	del	NA	NA	NA	NA	NA	7	1	1	1	0.008151	12	1	3	1	0.000532	up	-	-	-	down	3.43E-01	2	0	0	2	0	156
	EDN3	del	NA	NA	NA	NA	NA	4	1	1	1	0.0455	11	2	3	1	0.019016	up	-	-	-	up	4.60E-06	17	5	0	3	9	131
	FAM210B	del	NA	NA	NA	NA	NA	4	1	0	1	0.0455	11	1	2	1	0.000911	up	-	-	-	down	2.65E-11	0	0	0	0	0	238
	HIBCH	del	5	1	0	1	0.083265	13	4	2	3	0.005211	19	5	4	4	0.030472	down	-	0.581584355	0.279116838	up	0.018715247	0	0	0	0	0	130
	NECAB3	del	NA	NA	NA	NA	NA	4	1	1	1	0.0455	13	1	4	1	0.000911	down	-	-	-	down	0.01130629	452	360	39	25	28	805
	RAB22A	del	NA	NA	NA	NA	NA	5	1	1	1	0.025347	14	2	3	1	0.008151	down	-	-	-	down	4.54971E-15	3	0	0	3	0	139
	RABGAP1L	amp	3	2	3	0	0.198543	8	6	6	0	0.040244	14	9	7	0	0.032708	up	0.069415063	0.018155053	0.013254126	up	3.89E-02	4	0	3	0	1	60
	TFAP2C	del	NA	NA	NA	NA	NA	5	1	0	1	0.025347	10	2	2	1	0.025347	up	-	-	-	down	0.030279695	1	0	0	1	0	162
	ZNF217	del	NA	NA	NA	NA	NA	7	1	1	1	0.008151	12	1	3	1	0.000532	up	-	-	-	down	0.541983042	0	0	0	0	0	80
	RPRD2	del	NA	NA	NA	NA	NA	10	1	4	1	0.001565	19	6	5	1	0.75867	up	-	-	-	down	0.52833752	0	0	0	0	0	92
	ARAP2	amp	1	3	1	0	0.157299	1	10	1	3	0.0027	5	18	1	4	0.385953	up	-	-	-	down	4.46E-01	0	0	0	0	0	62
	FAM107B	amp	1	3	1	0	0.083265	3	7	2	2	0.001863	6	9	3	4	0.111841	down	-	0.002728927	0.142992319	up	3.89E-05	0	0	0	0	0	41
	MYH9	amp	1	2	1	0	0.157299	1	12	1	5	0.033756	4	20	2	7	0.054559	up	-	-	0.12895282	up	0.044067039	8	7	0	0	1	69
DiseaseFree Survival	COL18A1	del	3	2	0	2	0.317311	7	12	0	6	0.109735	8	16	0	9	0.026398	down	0.32104879	0.16617485	0.278565636	down	2.77E-05	138	87	29	17	5	503
	PAK3	amp	6	1	3	1	0.248055	8	2	4	1	0.149196	11	3	6	1	0.044667	down	6.40316E-09	0.268075704	0.83121386	up	0.000980777	0	0	0	0	0	69
	POLA1	del	1	6	1	3	0.257602	2	9	1	5	0.105446	4	12	1	7	0.025183	down	0.946142271	0.629805696	0.778146707	down	1.47253E-14	1	0	0	0	1	41
	STS	del	3	5	1	4	0.105084	3	10	1	6	0.111952	6	12	1	7	0.031424	down	4.08461E-05	0.061404447	0.04774319	up	0.397982858	1	0	1	0	0	52
	GALM	del	NA	NA	NA	NA	NA	NA	NA	NA	NA	NA	18	1	4	1	0.000108	down	-	-	-	up	6.2202E-05	0	0	0	0	0	54
	PADI1	amp	NA	NA	NA	NA	NA	1	13	1	6	0.05523	1	16	1	7	0.027365	down	-	-	-	down	0.003183165	0	0	0	0	0	44
	PPM1F	amp	NA	NA	NA	NA	NA	NA	NA	NA	NA	NA	1	17	1	5	0.008135	down	-	-	-	down	0.061909519	0	0	0	0	0	47
	SRSF7	del	NA	NA	NA	NA	NA	NA	NA	NA	NA	NA	14	1	3	1	0.000911	down	-	-	-	down	7.4512E-09	83	68	11	4	0	233
	ACSL4	amp	3	2	2	1	0.198543	10	4	6	1	0.070335	10	4	6	1	0.070335	up	0.000119636	0.268728976	0.268728976	down	1.05895E-09	0	0	0	0	0	24
	CHCHD10	amp	NA	NA	NA	NA	NA	NA	NA	NA	NA	NA	NA	NA	NA	NA	NA	up	-	-	-	up	0.000450521	8	8	0	0	0	112
	CHRDL1	amp	6	2	4	1	0.157939	10	3	6	1	0.080727	12	3	7	1	0.077366	down	0.462817821	0.851178094	0.578505123	up	0.046342875	158	120	29	9	0	205
	FAM19A5	amp	NA	NA	NA	NA	NA	NA	NA	NA	NA	NA	NA	NA	NA	NA	NA	down	-	-	-	down	2.93674E-09	3	2	1	0	0	185
	FAM72D	del	NA	NA	NA	NA	NA	12	1	4	1	0.179433	19	5	5	1	0.450035	down	-	-	-	down	9.21059E-17	1183	940	112	89	42	1640
	FCGR3A	del	7	1	3	1	0.584577	11	1	5	1	0.444233	16	3	6	1	0.646467	down	-	-	-	down	1.16013E-05	2	0	2	0	0	61
	GPR143	del	2	4	1	2	0.445243	4	7	1	4	0.090709	4	9	1	5	0.075774	down	0.310704486	0.058967965	0.19347861	down	1.02966E-22	0	0	0	0	0	32
	GPR89A	del	NA	NA	NA	NA	NA	NA	NA	NA	NA	NA	18	3	4	0	0.563703	down	-	-	-	down	0.002497993	42	30	12	0	0	373
	HS6ST2	amp	6	2	3	1	0.195995	13	3	7	1	0.078336	13	3	7	1	0.078336	up	0.633163468	0.7960648	0.7960648	down	7.4156E-29	0	0	0	0	0	54
	LANCL3	del	2	5	2	2	0.8849	3	7	2	3	0.511854	4	9	2	5	0.256235	down	0.02765389	0.067485848	0.011686205	up	0.001437329	9	1	5	0	3	42
	PDZK1	del	NA	NA	NA	NA	NA	NA	NA	NA	NA	NA	18	4	5	0	0.508815	down	-	-	-	up	0.251718825	0	0	0	0	0	215
	POLR3C	del	NA	NA	NA	NA	NA	NA	NA	NA	NA	NA	17	4	5	0	0.50255	down	-	-	-	down	1.5718E-13	63	37	22	2	2	112
	RPA4	amp	7	3	4	2	0.553137	9	5	5	3	0.604256	11	5	7	3	0.351166	up	0.881282265	0.611952411	0.89887041	down	0.000235412	0	0	0	0	0	46
	RPS6KA6	amp	5	2	2	1	0.343067	10	5	6	2	0.14511	10	7	6	3	0.106559	up	0.497587374	0.714241758	0.714241758	up	0.000293504	0	0	0	0	0	24
	TLR8	del	2	3	1	1	0.563703	4	7	1	3	0.108062	5	9	1	4	0.065411	down	-	0.560689158	0.546696013	up	0.142191815	286	242	22	16	6	279
	TMEM204	del	NA	NA	NA	NA	NA	4	1	1	0	1	9	2	2	0	0.405381	down	-	-	-	down	0.007540592	0	0	0	0	0	24
	TSC22D3	amp	4	2	2	1	0.225253	8	4	5	1	0.061159	9	5	6	1	0.063164	up	0.463598404	0.21552315	0.370726392	up	0.004237511	10	2	0	0	8	33

2.7 Table S7. Genes that significantly distinguished the amp and del groups by at least one threshold

DFS (31 genes)				
GK5	CASP5	IGF2	RBFOX1	ZNF630
C20orf144	C20orf85	CASS4	CTSZ	CWF19L2
DOK5	EDN3	FAM210B	HIBCH	NECAB3
RAB22A	RABGAP1L	TFAP2C	ZNF217	RPRD2
ARAP2	FAM107B	MYH9	COL18A1	PAK3
POLA1	STS	GALM	PADI1	PPM1F
SRSF7				

OS (6 genes)				
GK5	ARAP2	RPRD2	DRD4	IFITM1
ZNF658				

2.8 Table S8. Meta-analysis using expression data from ONCOMINE

							Cohort														Expression	
**Survial**	**larkerNam**	**CN**	**Exprs**	**Weight**	**Zscore**	**P-value**	**Alon**	**Gaedcke**	**Gaspar**	**Graudens**	**Kaiser**	**Ki**	**Kurashina**	**Notterman**	**batesBellv**	**Skrzypczak**	**krzypczak**	**TCGA**	**TCGA2**	**Zou**	**+**	**-**
	DRD4	Amp	up	405	2.15	0.03156	?	+	?	?	+	?	?	+	-	-	+	+	-	?	5	3
	ZNF658	Del	dw	300	2.166	0.0303	?	?	+	?	+	-	+	?	-	-	-	?	+	?	4	4
**OS**	GK5	Del	dw	546	6.374	1.85E-10	?	+	?	?	+	?	-	?	+	+	+	+	+	?	7	1
	RPRD2	Del	dw	611	5.371	7.85E-08	?	-	+	?	-	?	+	?	+	+	+	0	+	0	6	2
	IFITM1	Del	dw	666	16.974	1.27E-64	+	+	+	?	+	+	?	+	+	+	+	+	-	+	11	1
	CWF19L2	Amp	up	563	2.684	0.007274	?	+	+	?	+	+	-	?	+	+	+	-	-	+	8	3
	MYH9	Amp	up	600	2.889	0.003866	+	-	+	-	-	+	+	+	+	+	+	+	0	?	9	3
	ARAP2	Amp	up	414	4.124	3.73E-05	?	-	-	?	+	?	0	?	+	+	+	+	-	?	5	3
	RABGAP1L	Amp	up	556	4.806	1.54E-06	?	0	-	?	+	?	+	?	+	+	-	-	+	+	6	3
	NECAB3	Del	dw	505	15.383	2.14E-53	?	+	?	?	+	?	+	-	+	+	+	+	+	?	8	1
	RAB22A	Del	dw	620	18.905	1.03E-79	?	+	-	+	+	?	+	?	+	+	+	+	+	+	10	1
	STS	Del	dw	642	3.763	0.000168	?	-	+	?	+	-	+	+	+	-	-	+	+	?	7	4
	GALM	Del	dw	546	2.551	0.01074	?	0	?	-	-	?	+	?	+	0	0	0	+	?	3	2
	HIBCH	Del	dw	679	3.835	0.000126	?	0	+	0	0	-	+	?	+	-	-	0	+	-	4	4
**DFS**	GK5	Del	dw	546	6.374	1.85E-10	?	+	?	?	+	?	-	?	+	+	+	+	+	?	7	1
	CTSZ	Del	dw	622	11.124	9.64E-29	?	0	+	?	-	+	+	+	-	-	+	0	+	?	6	3
	C20orf144	Del	dw	397	12.826	1.17E-37	?	-	?	?	+	?	+	?	-	+	+	?	+	?	5	2
	COL18A1	Del	dw	651	8.517	1.64E-17	?	+	+	?	+	+	-	+	-	+	+	+	0	?	8	2
	ZNF630	Del	dw	498	5.414	6.18E-08	?	+	?	?	-	?	+	?	+	+	-	+	+	?	6	2
	POLA1	Del	dw	555	13.338	1.39E-40	?	+	?	?	+	?	+	+	+	+	+	+	+	?	9	0
	C20orf85	Del	dw	507	15.717	1.16E-55	?	+	?	?	-	?	+	?	+	+	+	+	+	+	8	1
	SRSF7	Del	dw	663	9.487	2.38E-21	?	+	+	+	+	-	+	-	+	+	+	+	+	?	10	2
	IGF2	Del	dw	679	8.624	6.45E-18	+	+	-	+	+	-	-	-	+	+	+	+	+	?	9	4
	CASS4	Del	dw	537	15.274	1.14E-52	?	+	?	?	+	?	+	?	-	+	+	+	+	?	7	1

2.9 Table S9. Performance of prediction models on the Chonnam-COAD test dataset

**Model**	**OS**				**DFS**			
	**AUC**	**F1**	**Prec**	**Sens**	**AUC**	**F1**	**Prec**	**Sens**
baseline	0.659	0.240	0.143	0.750	0.681	0.286	0.175	0.786
1	0.690	0.258	0.211	0.333	0.672	0.282	0.172	0.786
2	0.633	0.246	0.151	0.667	0.632	0.306	0.190	0.786
3	0.656	0.234	0.138	0.750	0.720	0.316	0.209	0.643
4	0.653	0.257	0.155	0.750	0.614	0.310	0.193	0.786
5	0.665	0.207	0.176	0.250	0.671	0.282	0.172	0.786
6	0.651	0.216	0.129	0.667	0.644	0.301	0.186	0.786
7	0.645	0.143	0.125	0.167	0.639	0.328	0.208	0.786

OS, 5-year overall survival; **DFS**, 5-year disease-free survival; **AUC**, area under the curve; **F1**, F1-score, $\frac{2*precision*recall}{precision+recall}$; **Prec**, precision, $\frac{\text{TP}}{\text{TP + FP}}$; **Sens**, sensitivity (recall), $\frac{\text{TP}}{\text{TP + FN}}$; **TP**, true positive; **FP**, false positive; **FN**, false negative.

2.10 Table S10. Performance of prediction models on a public dataset (GSE17536)

Model	OS				DFS			
	**AUC**	**F1**	**Prec**	**Sens**	**AUC**	**F1**	**Prec**	**Sens**
baseline	0.763	0.571	0.821	0.438	0.544	0.364	0.250	0.667
1	0.765	0.472	0.758	0.342	0.572	0.355	0.250	0.611
2	0.745	0.523	0.824	0.384	0.493	0.326	0.226	0.583
3	0.752	0.238	0.909	0.137	0.546	0.336	0.236	0.583
4	0.748	0.427	0.733	0.301	0.556	0.325	0.230	0.556
5	0.753	0.420	0.778	0.288	0.571	0.355	0.250	0.611
6	0.736	0.383	0.857	0.247	0.504	0.299	0.209	0.528
7	0.727	0.337	0.727	0.219	0.556	0.328	0.233	0.556

OS, 5-year overall survival; **DFS**, 5-year disease-free survival; **AUC**, area under the curve; **F1**, F1-score, $\frac{2*precision*recall}{precision+recall}$; **Prec**, precision, $\frac{\mathrm{TP}}{\mathrm{TP + FP}}$; **Sens**, sensitivity (recall), $\frac{\mathrm{TP}}{\mathrm{TP + FN}}$; **TP**, true positive; **FP**, false positive; **FN**, false negative.

2.11 Table S11. Performance of prediction models on a public dataset (GSE17537)

Model	OS				DFS			
	**AUC**	**F1**	**Prec**	**Sens**	**AUC**	**F1**	**Prec**	**Sens**
baseline	0.766	0.571	0.444	0.800	0.949	0.691	0.528	1.000
1	0.796	0.698	0.652	0.750	0.944	0.704	0.543	1.000
2	0.774	0.638	0.556	0.750	0.942	0.704	0.543	1.000
3	0.753	0.667	0.684	0.650	0.963	0.704	0.543	1.000
4	0.790	0.647	0.786	0.550	0.942	0.717	0.559	1.000
5	0.770	0.686	0.800	0.600	0.959	0.691	0.528	1.000
6	0.767	0.650	0.650	0.650	0.953	0.704	0.543	1.000
7	0.769	0.686	0.800	0.600	0.947	0.704	0.543	1.000

OS, 5-year overall survival; **DFS**, 5-year disease-free survival; **AUC**, area under the curve; **F1**, F1-score, $\frac{2*precision*recall}{precision+recall}$; **Prec**, precision, $\frac{\mathrm{TP}}{\mathrm{TP + FP}}$; **Sens**, sensitivity (recall), $\frac{\mathrm{TP}}{\mathrm{TP + FN}}$; **TP**, true positive; **FP**, false positive; **FN**, false negative.

3. Supplementary Figures

3.1 Supplementary Figure S1.

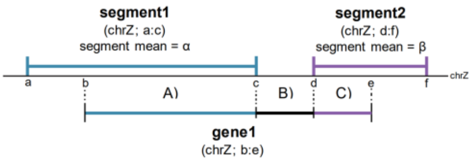

Supplementary Figure S1. An example of calculating a gene value when multiple segments are annotated to one gene An example of calculating a gene value when multiple segments are annotated to one gene. A) represents a region where gene 1 and segment 1 overlap on chromosome Z, B) represents a space between segment 1 and segment 2, and C) represents a region where gene 1 and segment 2 overlap. We assume that chromosome Z has segment 1 with a start position of a and an end position of c and segment 2 with a start position of d and end position f. Assuming that gene 1 is located at b:e of chromosome A, the value of gene 1 is calculated as follows: A) The ratio of segment 1 to gene 1 is (c-b). This is multiplied by the segment 1 mean value of α, B) which is the space between segments 1 and 2 and has the average value of the adjacent segments, segment 1 and segment 2. C) is calculated in the same manner as A). Gene 1 is calculated as the sum of values A), B), and C). The formula is shown in the equation below.

$$gene1 = \frac{1}{e-b}\left((c-b)\cdot\alpha + (d-c)\cdot\frac{\alpha+\beta}{2} + (e-d)\cdot\beta\right)$$

3.2 Supplementary Figure S2.

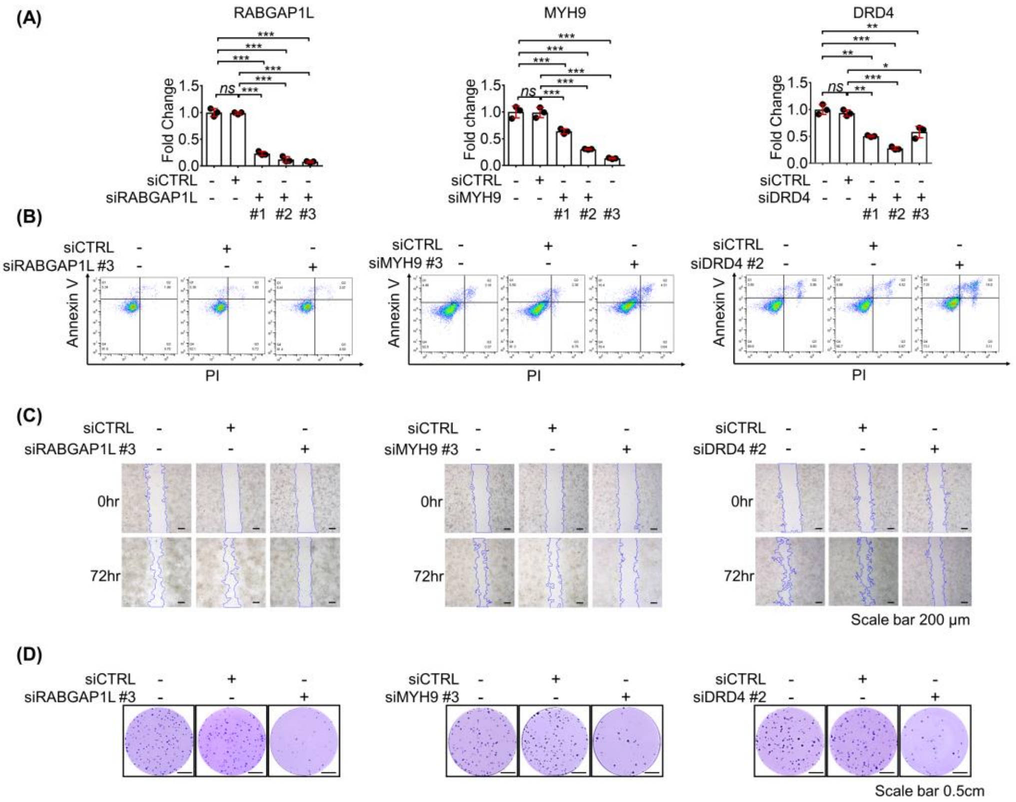

Supplementary Figure S3. Verification of the protumor activity of the three genes. (A) The gene silencing efficiency of three siRNAs targeting each of the three genes was assessed by qPCR to choose the most efficient siRNA sequence. (B) FACS analysis using Annexin V staining was performed to assess the effects of each gene knockdown on apoptosis. (C) The effect of the knockdown of each gene on cell migration was assessed by a wound healing assay. (D) Clonogenic assays were performed to evaluate the effect of the knockdown of each gene on cell survival.

References

1. Amgalan B, Lee H. DEOD: uncovering dominant effects of cancer-driver genes based on a partial covariance selection method. Bioinformatics. 2015; 31: 2452-60.

2. Tan R, Wang Y, Kleinstein SE, Liu Y, Zhu X, Guo H, et al. An evaluation of copy number variation detection tools from whole-exome sequencing data. Human mutation. 2014; 35: 899-907.

3. Wang Y, Klijn JG, Zhang Y, Sieuwerts AM, Look MP, Yang F, et al. Gene-expression profiles to predict distant metastasis of lymph-node-negative primary breast cancer. The Lancet. 2005; 365: 671-9.

4. Sim W, Lee J, Choi C. Robust method for identification of prognostic gene signatures from gene expression profiles. Scientific reports. 2017; 7: 1-11.

5. Alon U, Barkai N, Notterman DA, Gish K, Ybarra S, Mack D, et al. Broad patterns of gene expression revealed by clustering analysis of tumor and normal colon tissues probed by oligonucleotide arrays. Proceedings of the National Academy of Sciences. 1999; 96: 6745-50.

6. Hu Y, Gaedcke J, Emons G, Beissbarth T, Grade M, Jo P, et al. Colorectal cancer susceptibility loci as predictive markers of rectal cancer prognosis after surgery. Genes, Chromosomes and Cancer. 2018; 57: 140-9.

7. Gaspar C, Cardoso J, Franken P, Molenaar L, Morreau H, Möslein G, et al. Cross-species comparison of human and mouse intestinal polyps reveals conserved mechanisms in adenomatous polyposis coli (APC)-driven tumorigenesis. The American journal of pathology. 2008; 172: 1363-80.

8. Graudens E, Boulanger V, Mollard C, Mariage-Samson R, Barlet X, Grémy G, et al. Deciphering cellular states of innate tumor drug responses. Genome biology. 2006; 7: 1-21.

9. Kaiser S, Park Y-K, Franklin JL, Halberg RB, Yu M, Jessen WJ, et al. Transcriptional recapitulation and subversion of embryonic colon development by mouse colon tumor models and human colon cancer. Genome biology. 2007; 8: 1-26.

10. Ki DH, Jeung HC, Park CH, Kang SH, Lee GY, Lee WS, et al. Whole genome analysis for liver metastasis gene signatures in colorectal cancer. International journal of cancer. 2007; 121: 2005-12.

11. Kurashina K, Yamashita Y, Ueno T, Koinuma K, Ohashi J, Horie H, et al. Chromosome copy number analysis in screening for prognosis-related genomic regions in colorectal carcinoma. Cancer science. 2008; 99: 1835-40.

12. Notterman DA, Alon U, Sierk AJ, Levine AJ. Transcriptional gene expression profiles of colorectal adenoma, adenocarcinoma, and normal tissue examined by oligonucleotide arrays. Cancer research. 2001; 61:

3124-30.

13. Sabates-Bellver J, Van der Flier LG, de Palo M, Cattaneo E, Maake C, Rehrauer H, et al. Transcriptome profile of human colorectal adenomas. Molecular cancer research. 2007; 5: 1263-75.

14. Skrzypczak M, Goryca K, Rubel T, Paziewska A, Mikula M, Jarosz D, et al. Modeling oncogenic signaling in colon tumors by multidirectional analyses of microarray data directed for maximization of analytical reliability. PloS one. 2010; 5: e13091.

15. Zou T-T, Selaru FM, Xu Y, Shustova V, Yin J, Mori Y, et al. Application of cDNA microarrays to generate a molecular taxonomy capable of distinguishing between colon cancer and normal colon. Oncogene. 2002; 21: 4855-62.

16. Network CGA. Comprehensive molecular characterization of human colon and rectal cancer. Nature. 2012; 487: 330.

17. Willer CJ, Li Y, Abecasis GR. METAL: fast and efficient meta-analysis of genomewide association scans. Bioinformatics. 2010; 26: 2190-1.
